# Supplementary material for: Epigenome-wide association data implicates DNA methylation-mediated genetic risk in psoriasis
Source: Clin Epigenetics. 2016 Dec 5;8:131. doi: 10.1186/s13148-016-0297-z (PMC5139011; doi:10.1186/s13148-016-0297-z)
Supplement: Additional file 1: — Table S1. Psoriasis-associated DMSs that are controlled by SNPs. Table S2 Disease severity and position of MethQTL CpGs. Table S3 The association between psoriasis and MethQTL-SNPs in MHC region. Table S4 The results of SNP-CpG pairs that mediate the genetic risk of psoriasis. Table S5 Cross validation between Illumina and Sequenom platform. (DOCX 112 kb) [file 13148_2016_297_MOESM1_ESM.docx]

**Table S1. Psoriasis-associated DMSs that are controlled by SNPs**

| SNP | | |  | CpG sites | | | | *P_value_* |
| --- | --- | --- | --- | --- | --- | --- | --- | --- |
| Chr | ID | Map |  | Chr | ID | Map | Gene_context |  |
| 6 | GA005301 | 31271640 |  | 1 | cg27571329 | 59859555 | Body;Body | 1.1E-09 |
| 6 | GA005301 | 31271640 |  | 19 | cg16651537 | 51226536 | TSS200 | 1.22E-09 |
| 6 | GA005301 | 31271640 |  | 11 | cg09061733 | 57364841 | TSS1500;TSS200 | 1.25E-09 |
| 6 | GA005301 | 31271640 |  | 2 | cg11354105 | 25475805 | Body;Body | 3.13E-09 |
| 6 | GA005301 | 31271640 |  | 12 | cg01856529 | 54653091 | 1stExon;5'UTR | 3.61E-09 |
| 6 | GA005301 | 31271640 |  | 15 | cg16085649 | 86186011 | Body;Body | 4.81E-09 |
| 6 | GA005301 | 31271640 |  | 16 | cg02550308 | 69310261 | Body | 4.88E-09 |
| 6 | GA005301 | 31271640 |  | 7 | cg09490124 | 105128488 | Body | 5.35E-09 |
| 6 | rs2853958 | 31261685 |  | 1 | cg09468264 | 59340913 | NA | 7.39E-09 |
| 6 | rs2394895 | 31239202 |  | 1 | cg09468264 | 59340913 | NA | 8.15E-09 |
| 6 | rs3132495 | 31261685 |  | 1 | cg09468264 | 59340913 | NA | 8.15E-09 |
| 6 | rs3134762 | 31261685 |  | 1 | cg09468264 | 59340913 | NA | 8.15E-09 |
| 6 | rs2394894 | 31239143 |  | 1 | cg09468264 | 59340913 | NA | 8.82E-09 |
| 6 | kgp1637562 | 31229552 |  | 1 | cg09468264 | 59340913 | NA | 8.85E-09 |
| 6 | rs2844626 | 31261775 |  | 1 | cg09468264 | 59340913 | NA | 8.85E-09 |
| 6 | rs746647 | 31261685 |  | 7 | cg09490124 | 105128488 | Body | 9.37E-09 |
| 6 | rs1265076 | 31145306 |  | 7 | cg09490124 | 105128488 | Body | 9.37E-09 |
| 6 | rs1265079 | 31144331 |  | 7 | cg09490124 | 105128488 | Body | 9.37E-09 |
| 6 | rs1265087 | 31142033 |  | 7 | cg09490124 | 105128488 | Body | 9.37E-09 |
| 6 | rs1265112 | 31150242 |  | 7 | cg09490124 | 105128488 | Body | 9.37E-09 |
| 6 | rs1265114 | 31149411 |  | 7 | cg09490124 | 105128488 | Body | 9.37E-09 |
| 6 | rs2517985 | 31151165 |  | 7 | cg09490124 | 105128488 | Body | 9.37E-09 |
| 6 | rs746647 | 31261685 |  | 11 | cg09061733 | 57364841 | TSS1500;TSS200 | 1.09E-08 |
| 6 | rs1265076 | 31145306 |  | 11 | cg09061733 | 57364841 | TSS1500;TSS200 | 1.09E-08 |
| 6 | rs1265079 | 31144331 |  | 11 | cg09061733 | 57364841 | TSS1500;TSS200 | 1.09E-08 |
| 6 | rs1265087 | 31142033 |  | 11 | cg09061733 | 57364841 | TSS1500;TSS200 | 1.09E-08 |
| 6 | rs1265112 | 31150242 |  | 11 | cg09061733 | 57364841 | TSS1500;TSS200 | 1.09E-08 |
| 6 | rs1265114 | 31149411 |  | 11 | cg09061733 | 57364841 | TSS1500;TSS200 | 1.09E-08 |
| 6 | rs2517985 | 31151165 |  | 11 | cg09061733 | 57364841 | TSS1500;TSS200 | 1.09E-08 |
| 6 | rs2395471 | 31272915 |  | 1 | cg09468264 | 59340913 | NA | 1.10E-08 |
| 6 | GA005301 | 31271640 |  | 6 | cg07207982 | 34984930 | Body | 1.21E-08 |
| 6 | GA005301 | 31271640 |  | 5 | cg05588228 | 145132641 | NA | 1.29E-08 |
| 6 | GA005301 | 31271640 |  | 3 | cg27405988 | 48171915 | NA | 1.37E-08 |
| 6 | GA005301 | 31271640 |  | 19 | cg03052794 | 908894 | Body | 1.50E-08 |
| 6 | rs2245822 | 31263023 |  | 1 | cg04087571 | 116723030 | Body | 1.6E-08 |
| 6 | rs10484554 | 31306778 |  | 1 | cg09468264 | 59340913 | NA | 1.66E-08 |
| 6 | rs2853952 | 31261685 |  | 1 | cg09468264 | 59340913 | NA | 1.66E-08 |
| 6 | rs9380238 | 31261685 |  | 1 | cg09468264 | 59340913 | NA | 1.80E-08 |
| 6 | kgp1637562 | 31229552 |  | 7 | cg09490124 | 105128488 | Body | 1.86E-08 |
| 6 | rs2844626 | 31261775 |  | 7 | cg09490124 | 105128488 | Body | 1.86E-08 |
| 6 | GA005301 | 31271640 |  | 9 | cg13670057 | 133721945 | Body;Body | 1.89E-08 |
| 6 | rs2853953 | 31261685 |  | 1 | cg09914444 | 46972183 | TSS1500 | 2.1E-08 |
| 6 | rs3132505 | 31261685 |  | 1 | cg09468264 | 59340913 | NA | 2.18E-08 |
| 6 | rs3132506 | 31261685 |  | 1 | cg09468264 | 59340913 | NA | 2.18E-08 |
| 6 | kgp1637562 | 31229552 |  | 11 | cg09061733 | 57364841 | TSS1500;TSS200 | 2.19E-08 |
| 6 | rs2844626 | 31261775 |  | 11 | cg09061733 | 57364841 | TSS1500;TSS200 | 2.19E-08 |
| 6 | rs2853958 | 31261685 |  | 17 | cg21653149 | 4108148 | Body;Body | 2.28E-08 |
| 6 | GA005301 | 31271640 |  | 20 | cg20918393 | 19867136 | NA | 2.34E-08 |
| 6 | GA005301 | 31271640 |  | 3 | cg01070987 | 149687002 | Body;Body | 2.35E-08 |
| 6 | rs2395471 | 31272915 |  | 7 | cg09490124 | 105128488 | Body | 2.55E-08 |
| 6 | rs2853952 | 31261685 |  | 11 | cg04087571 | 116723030 | Body | 2.6E-08 |
| 6 | rs10484554 | 31306778 |  | 11 | cg09061733 | 57364841 | TSS1500;TSS200 | 2.57E-08 |
| 6 | rs10484554 | 31306778 |  | 19 | cg09914444 | 46972183 | TSS1500 | 2.6E-08 |
| 6 | rs2395471 | 31272915 |  | 11 | cg09061733 | 57364841 | TSS1500;TSS200 | 2.59E-08 |
| 6 | rs2853952 | 31261685 |  | 19 | cg19430423 | 41702153 | Body | 2.59E-08 |
| 6 | rs4406273 | 31261685 |  | 1 | cg09468264 | 59340913 | NA | 2.61E-08 |
| 6 | rs2853958 | 31261685 |  | 11 | cg09061733 | 57364841 | TSS1500;TSS200 | 2.64E-08 |
| 6 | rs1265076 | 31145306 |  | 1 | cg09468264 | 59340913 | NA | 2.67E-08 |
| 6 | rs1265079 | 31144331 |  | 1 | cg09468264 | 59340913 | NA | 2.67E-08 |
| 6 | rs1265087 | 31142033 |  | 1 | cg09468264 | 59340913 | NA | 2.67E-08 |
| 6 | rs1265112 | 31150242 |  | 1 | cg09468264 | 59340913 | NA | 2.67E-08 |
| 6 | rs1265114 | 31149411 |  | 1 | cg09468264 | 59340913 | NA | 2.67E-08 |
| 6 | rs2517985 | 31151165 |  | 1 | cg09468264 | 59340913 | NA | 2.67E-08 |
| 6 | rs746647 | 31261685 |  | 1 | cg09468264 | 59340913 | NA | 2.67E-08 |
| 6 | rs2844626 | 31261775 |  | 1 | cg21116284 | 92216881 | Body | 2.71E-08 |
| 6 | kgp1637562 | 31229552 |  | 1 | cg21116284 | 92216881 | Body | 2.71E-08 |
| 6 | GA005301 | 31271640 |  | 1 | cg09468264 | 59340913 | NA | 2.77E-08 |
| 6 | GA005301 | 31271640 |  | 10 | cg19730379 | 104542002 | Body;Body | 2.83E-08 |
| 6 | rs10484554 | 31306778 |  | 12 | cg09043214 | 6442873 | Body | 2.87E-08 |
| 6 | rs2853952 | 31261685 |  | 12 | cg09043214 | 6442873 | Body | 2.87E-08 |
| 6 | GA005301 | 31271640 |  | 4 | cg02616418 | 154125681 | 5'UTR;1stExon | 3.04E-08 |
| 6 | rs746647 | 31261685 |  | 15 | cg16085649 | 86186011 | Body;Body | 3.09E-08 |
| 6 | rs1265076 | 31145306 |  | 15 | cg16085649 | 86186011 | Body;Body | 3.09E-08 |
| 6 | rs1265079 | 31144331 |  | 15 | cg16085649 | 86186011 | Body;Body | 3.09E-08 |
| 6 | rs1265087 | 31142033 |  | 15 | cg16085649 | 86186011 | Body;Body | 3.09E-08 |
| 6 | rs1265112 | 31150242 |  | 15 | cg16085649 | 86186011 | Body;Body | 3.09E-08 |
| 6 | rs1265114 | 31149411 |  | 15 | cg16085649 | 86186011 | Body;Body | 3.09E-08 |
| 6 | rs2517985 | 31151165 |  | 15 | cg16085649 | 86186011 | Body;Body | 3.09E-08 |
| 6 | rs2853953 | 31261685 |  | 19 | cg06834507 | 200876957 | Body | 3.2E-08 |
| 6 | rs2844626 | 31261775 |  | 12 | cg01856529 | 54653091 | 1stExon;5'UTR | 3.20E-08 |
| 6 | kgp1637562 | 31229552 |  | 12 | cg01856529 | 54653091 | 1stExon;5'UTR | 3.20E-08 |
| 4 | rs2719947 | 42417138 |  | 1 | cg21775279 | 28285385 | TSS1500;3'UTR | 3.21E-08 |
| 6 | GA005301 | 31271640 |  | 17 | cg21653149 | 4108148 | Body;Body | 3.23E-08 |
| 6 | rs2853953 | 31261685 |  | 11 | cg09061733 | 57364841 | TSS1500;TSS200 | 3.24E-08 |
| 6 | rs2395471 | 31272915 |  | 12 | cg01856529 | 54653091 | 1stExon;5'UTR | 3.37E-08 |
| 6 | rs2245822 | 31263023 |  | 19 | cg19430423 | 41702153 | Body | 3.42E-08 |
| 6 | rs2853953 | 31261685 |  | 12 | cg04087571 | 116723030 | Body | 3.7E-08 |
| 6 | rs13203895 | 31276305 |  | 1 | cg04087571 | 116723030 | Body | 3.8E-08 |
| 6 | kgp12175980 | 31144960 |  | 1 | cg09468264 | 59340913 | NA | 3.81E-08 |
| 6 | rs10484554 | 31306778 |  | 1 | cg20335425 | 153363264 | 5'UTR | 4.03E-08 |
| 6 | rs2853952 | 31261685 |  | 1 | cg20335425 | 153363264 | 5'UTR | 4.03E-08 |
| 6 | GA005301 | 31271640 |  | 16 | cg27456203 | 69924616 | Body;Body | 4.07E-08 |
| 6 | rs2245822 | 31263023 |  | 12 | cg09914444 | 46972183 | TSS1500 | 4.1E-08 |
| 6 | GA005301 | 31271640 |  | 12 | cg03188064 | 122096667 | Body | 4.15E-08 |
| 6 | rs4406273 | 31261685 |  | 11 | cg09061733 | 57364841 | TSS1500;TSS200 | 4.16E-08 |
| 6 | rs2245822 | 31263023 |  | 11 | cg09061733 | 57364841 | TSS1500;TSS200 | 4.33E-08 |
| 6 | rs4406273 | 31261685 |  | 19 | cg19430423 | 41702153 | Body | 4.38E-08 |
| 6 | rs746647 | 31261685 |  | 1 | cg27571329 | 59859555 | Body;Body | 4.83E-08 |
| 6 | rs1265076 | 31145306 |  | 1 | cg27571329 | 59859555 | Body;Body | 4.83E-08 |
| 6 | rs1265079 | 31144331 |  | 1 | cg27571329 | 59859555 | Body;Body | 4.83E-08 |
| 6 | rs1265087 | 31142033 |  | 1 | cg27571329 | 59859555 | Body;Body | 4.83E-08 |
| 6 | rs1265112 | 31150242 |  | 1 | cg27571329 | 59859555 | Body;Body | 4.83E-08 |
| 6 | rs1265114 | 31149411 |  | 1 | cg27571329 | 59859555 | Body;Body | 4.83E-08 |
| 6 | rs2517985 | 31151165 |  | 1 | cg27571329 | 59859555 | Body;Body | 4.83E-08 |
| 6 | rs13203895 | 31276305 |  | 19 | cg06834507 | 200876957 | Body;Body | 4.8E-08 |
| 6 | rs2853953 | 31261685 |  | 1 | cg20335425 | 153363264 | 5'UTR | 4.88E-08 |
| 6 | rs13203895 | 31276305 |  | 12 | cg09914444 | 46972183 | TSS1500 | 5.1E-08 |
| 6 | GA005301 | 31271640 |  | 13 | cg27518324 | 99630505 | Body;TSS200 | 5.26E-08 |
| 6 | kgp12175980 | 31144960 |  | 19 | cg19430423 | 41702153 | Body | 5.37E-08 |
| 6 | rs10484554 | 31306778 |  | 18 | cg04087571 | 116723030 | Body | 5.6E-08 |
| 6 | rs2853952 | 31261685 |  | 18 | cg21261487 | 74825893 | 5'UTR;5'UTR | 5.56E-08 |
| 6 | rs2844627 | 31261685 |  | 1 | cg09468264 | 59340913 | NA | 5.59E-08 |
| 6 | rs746647 | 31261685 |  | 17 | cg21653149 | 4108148 | Body;Body | 5.79E-08 |
| 6 | rs1265076 | 31145306 |  | 17 | cg21653149 | 4108148 | Body;Body | 5.79E-08 |
| 6 | rs1265079 | 31144331 |  | 17 | cg21653149 | 4108148 | Body;Body | 5.79E-08 |
| 6 | rs1265087 | 31142033 |  | 17 | cg21653149 | 4108148 | Body;Body | 5.79E-08 |
| 6 | rs1265112 | 31150242 |  | 17 | cg21653149 | 4108148 | Body;Body | 5.79E-08 |
| 6 | rs1265114 | 31149411 |  | 17 | cg21653149 | 4108148 | Body;Body | 5.79E-08 |
| 6 | rs2517985 | 31151165 |  | 17 | cg21653149 | 4108148 | Body;Body | 5.79E-08 |
| 6 | GA005301 | 31271640 |  | 12 | cg03665360 | 54052859 | NA | 5.86E-08 |
| 6 | GA005301 | 31271640 |  | 5 | cg12198841 | 115941397 | NA | 5.86E-08 |
| 6 | rs746647 | 31261685 |  | 16 | cg02550308 | 69310261 | Body | 5.94E-08 |
| 6 | rs1265076 | 31145306 |  | 16 | cg02550308 | 69310261 | Body | 5.94E-08 |
| 6 | rs1265079 | 31144331 |  | 16 | cg02550308 | 69310261 | Body | 5.94E-08 |
| 6 | rs1265087 | 31142033 |  | 16 | cg02550308 | 69310261 | Body | 5.94E-08 |
| 6 | rs1265112 | 31150242 |  | 16 | cg02550308 | 69310261 | Body | 5.94E-08 |
| 6 | rs1265114 | 31149411 |  | 16 | cg02550308 | 69310261 | Body | 5.94E-08 |
| 6 | rs2517985 | 31151165 |  | 16 | cg02550308 | 69310261 | Body | 5.94E-08 |
| 6 | rs2853958 | 31261685 |  | 8 | cg00288598 | 141555295 | Body;Body | 5.95E-08 |

SNP_ID: Based on Illumina HumanOmniZhonghua genotyping chip. CpGsites_ID: the Illumina CpG identifier based on illumina 450K methylation array. Chromosome number (Chr) and genomic coordinate_37 (Map information) of the interrogated probe. Gene_context: Location of the gene associated CpG-site(s) with respect to the gene context. *P*_value_ is calculated under an additive model test between DNA methylation and Whole genome genotypes. The table is sorted by decreasing statistic significance. NA: not available.

**Table S2. Disease severity and position of MethQTL CpGs**

| PASI | Sample number | Average PASI | Position | | |
| --- | --- | --- | --- | --- | --- |
|  |  |  | Promoter | Gene body | Intergenic |
| <5 | 81 | 2.43 | 0.5985 | 0.5747 | 0.6273 |
| 5~10 | 25 | 6.74 | 0.6108 | 0.5804 | 0.6276 |
| >10 | 8 | 12.3 | 0.6281 | 0.5895 | 0.6187 |

**Table S3. The association between psoriasis and MethQTL-SNPs in MHC region.**

| SNP | CHR | Map | Allele_A | Allele_B | Genotype count | Call | *P_HWE_* | Dominant | |  | Additive | |  | Recessive | |
| --- | --- | --- | --- | --- | --- | --- | --- | --- | --- | --- | --- | --- | --- | --- | --- |
|  |  |  |  |  | (AA/AB/BB) | rate |  | OR | *P* |  | OR | *P* |  | OR | *P* |
| kgp12175980 | 6 | 31112737 | A | C | 10/92/73 | 0.99 | 0.01 | 15.31 | 5.31E-12 |  | 9.14 | 1.02E-09 |  | 1.27 | 7.40E-01 |
| rs4406273 | 6 | 31266090 | A | G | 11/94/72 | 1 | 0.01 | 15.08 | 3.25E-12 |  | 8 | 2.81E-09 |  | 0.94 | 9.24E-01 |
| rs2853958 | 6 | 31233074 | A | C | 7/92/71 | 0.96 | 0.01 | 13.81 | 1.89E-11 |  | 8.52 | 3.16E-09 |  | 0.74 | 6.95E-01 |
| rs2245822 | 6 | 31230800 | A | G | 13/93/70 | 0.99 | 0.02 | 14.34 | 4.91E-12 |  | 7.61 | 4.17E-09 |  | 1.24 | 7.27E-01 |
| rs10484554 | 6 | 31274555 | A | G | 13/93/71 | 1 | 0.03 | 13.71 | 8.00E-12 |  | 7.34 | 6.15E-09 |  | 1.23 | 7.38E-01 |
| rs2853952 | 6 | 31235869 | G | A | 13/93/71 | 1 | 0.03 | 13.71 | 8.00E-12 |  | 7.34 | 6.15E-09 |  | 1.23 | 7.38E-01 |
| rs2853953 | 6 | 31235505 | A | G | 13/92/71 | 0.99 | 0.03 | 13.57 | 9.95E-12 |  | 7.26 | 7.41E-09 |  | 1.24 | 7.27E-01 |
| rs13203895 | 6 | 31244082 | A | G | 13/91/70 | 0.98 | 0.03 | 13.14 | 1.94E-11 |  | 7.02 | 1.33E-08 |  | 1.23 | 7.37E-01 |
| rs1265076 | 6 | 31113083 | A | G | 15/96/66 | 1 | 0.02 | 13.66 | 6.39E-12 |  | 6.53 | 1.82E-08 |  | 1.09 | 8.86E-01 |
| rs1265079 | 6 | 31112108 | A | C | 15/96/66 | 1 | 0.02 | 13.66 | 6.39E-12 |  | 6.53 | 1.82E-08 |  | 1.09 | 8.86E-01 |
| rs1265087 | 6 | 31109810 | A | G | 15/96/66 | 1 | 0.02 | 13.66 | 6.39E-12 |  | 6.53 | 1.82E-08 |  | 1.09 | 8.86E-01 |
| rs1265112 | 6 | 31118019 | G | A | 15/96/66 | 1 | 0.02 | 13.66 | 6.39E-12 |  | 6.53 | 1.82E-08 |  | 1.09 | 8.86E-01 |
| rs1265114 | 6 | 31117188 | A | G | 15/96/66 | 1 | 0.02 | 13.66 | 6.39E-12 |  | 6.53 | 1.82E-08 |  | 1.09 | 8.86E-01 |
| rs2517985 | 6 | 31118942 | G | A | 15/96/66 | 1 | 0.02 | 13.66 | 6.39E-12 |  | 6.53 | 1.82E-08 |  | 1.09 | 8.86E-01 |
| rs746647 | 6 | 31114182 | G | A | 15/96/66 | 1 | 0.02 | 13.66 | 6.39E-12 |  | 6.53 | 1.82E-08 |  | 1.09 | 8.86E-01 |
| kgp1637562 | 6 | 31229552 | T | A | 32/82/62 | 0.99 | 0.64 | 13.95 | 6.71E-12 |  | 5.16 | 4.22E-08 |  | 2.14 | 9.83E-02 |
| rs2844626 | 6 | 31229552 | T | A | 32/82/62 | 0.99 | 0.64 | 13.95 | 6.71E-12 |  | 5.16 | 4.22E-08 |  | 2.14 | 9.83E-02 |
| rs2844627 | 6 | 31229462 | A | G | 26/80/70 | 0.99 | 0.75 | 13.43 | 1.26E-11 |  | 5.46 | 5.11E-08 |  | 1.93 | 1.84E-01 |
| rs2394895 | 6 | 31206979 | G | A | 26/96/55 | 1 | 0.16 | 10.49 | 4.27E-10 |  | 5.28 | 9.34E-08 |  | 2.55 | 7.51E-02 |
| rs3132495 | 6 | 31208881 | A | T | 26/96/55 | 1 | 0.16 | 10.49 | 4.27E-10 |  | 5.28 | 9.34E-08 |  | 2.55 | 7.51E-02 |
| rs3134762 | 6 | 31210866 | G | A | 26/96/55 | 1 | 0.16 | 10.49 | 4.27E-10 |  | 5.28 | 9.34E-08 |  | 2.55 | 7.51E-02 |
| rs2394894 | 6 | 31206920 | G | A | 26/95/55 | 0.99 | 0.17 | 10.39 | 5.14E-10 |  | 5.23 | 1.04E-07 |  | 2.57 | 7.19E-02 |
| rs3132505 | 6 | 31177503 | A | C | 26/97/54 | 1 | 0.12 | 9.797 | 1.16E-09 |  | 5.04 | 1.70E-07 |  | 2.55 | 7.51E-02 |
| rs3132506 | 6 | 31176226 | G | A | 26/97/54 | 1 | 0.12 | 9.797 | 1.16E-09 |  | 5.04 | 1.70E-07 |  | 2.55 | 7.51E-02 |
| GA005301 | 6 | 31239417 | A | G | 26/95/56 | 1 | 0.22 | 13.02 | 2.83E-11 |  | 4.58 | 4.32E-07 |  | 1.25 | 6.23E-01 |
| rs9380238 | 6 | 31267618 | G | A | 30/96/51 | 1 | 0.22 | 9.231 | 4.12E-09 |  | 4.07 | 1.42E-06 |  | 1.96 | 1.46E-01 |
| rs2395471 | 6 | 31240692 | G | A | 31/73/73 | 1 | 0.11 | 0.398 | 6.80E-03 |  | 0.36 | 1.87E-05 |  | 0.13 | 4.68E-06 |
| rs2719947 | 4 | 42384915 | A | G | 1/17/159 | 1 | 0.4 | 10.58 | 2.36E-02 |  | 10.09 | 2.57E-02 |  | 88 | 9.99E-01 |

*P_HWE_:* Hardy-Weinberg Equilibrium for each SNP. No deviation was observed (*P* > 0.001). OR: odds ratio. The association tests were performed under dominant, additive and recessive model respectively. Additive model highlighted in grey is used for illumination in main context.

**Table S4. The results of SNP-CpG pairs that mediate the genetic risk of psoriasis**

| **Psoriasis associated DMSs** | | | | | |  | **SNPs associated with DMSs** | | | | | | |
| --- | --- | --- | --- | --- | --- | --- | --- | --- | --- | --- | --- | --- | --- |
| Illumina ID | Chr | Gene context | Gene name | *P* value  (Meth vs. Pheno) | Beta difference |  | SNP | Map | *P* value (Geno vs. Pheno) | *P* value (Meth vs. Geno) | Adjusted *P* value (Meth vs. Geno) | Adjusted independent *P* value (Geno vs. Pheno) | CIT *P* |
| cg04087571 | 1 | Body | SIK3 | 8.41E-09 | -0.11 |  | rs13203895 | 31276305 | 1.33E-08 | 3.776E-08 | 0.022 | 0.004 | 0.022 |
| cg04087571 | 12 | Body | SIK3 | 8.41E-09 | -0.11 |  | rs2853953 | 31267728 | 7.41E-09 | 3.65E-08 | 0.024 | 0.01 | 0.024 |
| cg04087571 | 18 | Body | SIK3 | 8.41E-09 | -0.11 |  | rs10484554 | 31306778 | 6.15E-09 | 5.562E-08 | 0.027 | 0.005 | 0.027 |
| cg04087571 | 11 | Body | SIK3 | 8.41E-09 | -0.11 |  | rs2853952 | 31268092 | 6.15E-09 | 2.572E-08 | 0.027 | 0.008 | 0.027 |
| cg04087571 | 1 | Body | SIK3 | 8.41E-09 | -0.11 |  | rs2245822 | 31263023 | 4.17E-09 | 1.617E-08 | 0.027 | 0.01 | 0.027 |
| cg09914444 | 12 | TSS1500 | DMBX1;DMBX1 | 7.52E-09 | -0.11 |  | rs13203895 | 31276305 | 1.33E-08 | 5.078E-08 | 0.034 | 0.027 | 0.034 |
| cg09914444 | 12 | TSS1500 | DMBX1;DMBX1 | 7.52E-09 | -0.11 |  | rs2245822 | 31263023 | 4.17E-09 | 4.136E-08 | 0.037 | 0.037 | 0.037 |
| cg09914444 | 19 | TSS1500 | DMBX1;DMBX1 | 7.52E-09 | -0.11 |  | rs10484554 | 31306778 | 6.15E-09 | 2.592E-08 | 0.042 | 0.03 | 0.042 |
| cg06834507 | 19 | Body;Body | C1orf106;C1orf106 | 6.91E-10 | -0.11 |  | rs13203895 | 31276305 | 1.33E-08 | 4.844E-08 | 0.046 | 0.005 | 0.046 |
| cg06834507 | 19 | Body | C1orf106;C1orf106 | 6.91E-10 | -0.11 |  | rs2853953 | 31267728 | 7.41E-09 | 3.171E-08 | 0.047 | 0.007 | 0.047 |
| cg09914444 | 1 | TSS1500 | DMBX1;DMBX1 | 7.52E-09 | -0.11 |  | rs2853953 | 31267728 | 7.41E-09 | 2.084E-08 | 0.041 | 0.047 | 0.047 |
| cg09468264 | 1 | NA | NA | 6.94E-12 | 0.14 |  | kgp1637562 | 31112737 | 4.22E-08 | 8.85E-09 | 0.053 | < 0.001 | 0.053 |
| cg09468264 | 1 | NA | NA | 6.94E-12 | 0.14 |  | rs2844626 | 31261775 | 4.22E-08 | 8.85E-09 | 0.053 | < 0.001 | 0.053 |
| cg09061733 | 11 | TSS1500;TSS200 | SERPING1;SERPING1 | 4.08E-11 | 0.11 |  | rs1265076 | 31145306 | 1.82E-08 | 1.09E-08 | 0.069 | < 0.001 | 0.069 |
| cg09061733 | 11 | TSS1500;TSS200 | SERPING1;SERPING1 | 4.08E-11 | 0.11 |  | rs1265079 | 31144331 | 1.82E-08 | 1.09E-08 | 0.069 | < 0.001 | 0.069 |
| cg09061733 | 11 | TSS1500;TSS200 | SERPING1;SERPING1 | 4.08E-11 | 0.11 |  | rs1265087 | 31142033 | 1.82E-08 | 1.09E-08 | 0.069 | 0.001 | 0.069 |
| cg09061733 | 11 | TSS1500;TSS200 | SERPING1;SERPING1 | 4.08E-11 | 0.11 |  | rs1265112 | 31150242 | 1.82E-08 | 1.09E-08 | 0.069 | 0.001 | 0.069 |
| cg09061733 | 11 | TSS1500;TSS200 | SERPING1;SERPING1 | 4.08E-11 | 0.11 |  | rs1265114 | 31149411 | 1.82E-08 | 1.09E-08 | 0.069 | 0.001 | 0.069 |
| cg09061733 | 11 | TSS1500;TSS200 | SERPING1;SERPING1 | 4.08E-11 | 0.11 |  | rs2517985 | 31151165 | 1.82E-08 | 1.09E-08 | 0.069 | < 0.001 | 0.069 |
| cg09061733 | 11 | TSS1500;TSS200 | SERPING1;SERPING1 | 4.08E-11 | 0.11 |  | rs746647 | 31146405 | 1.82E-08 | 1.09E-08 | 0.069 | < 0.001 | 0.069 |
| cg01856529 | 12 | 1stExon;5'UTR | CBX5;CBX5;CBX5;CBX5 | 6.27E-10 | 0.11 |  | kgp1637562 | 31229552 | 4.22E-08 | 3.20E-08 | 0.071 | < 0.001 | 0.071 |
| cg01856529 | 12 | 1stExon;5'UTR | CBX5;CBX5;CBX5;CBX5 | 6.27E-10 | 0.11 |  | rs2844626 | 31261775 | 4.22E-08 | 3.20E-08 | 0.071 | < 0.001 | 0.071 |
| cg09061733 | 11 | TSS1500;TSS200 | SERPING1;SERPING1 | 4.08E-11 | 0.11 |  | kgp1637562 | 31229552 | 4.22E-08 | 2.19E-08 | 0.081 | 0.002 | 0.081 |
| cg09061733 | 11 | TSS1500;TSS200 | SERPING1;SERPING1 | 4.08E-11 | 0.11 |  | rs2844626 | 31261775 | 4.22E-08 | 2.19E-08 | 0.081 | < 0.001 | 0.081 |
| cg09490124 | 7 | Body | PUS7 | 3.50E-11 | 0.11 |  | rs1265076 | 31145306 | 1.82E-08 | 9.37E-09 | 0.085 | 0.001 | 0.085 |
| cg09490124 | 7 | Body | PUS7 | 3.50E-11 | 0.11 |  | rs1265087 | 31142033 | 1.82E-08 | 9.37E-09 | 0.085 | < 0.001 | 0.085 |
| cg09490124 | 7 | Body | PUS7 | 3.50E-11 | 0.11 |  | rs1265112 | 31150242 | 1.82E-08 | 9.37E-09 | 0.085 | < 0.001 | 0.085 |
| cg09490124 | 7 | Body | PUS7 | 3.50E-11 | 0.11 |  | rs1265114 | 31149411 | 1.82E-08 | 9.37E-09 | 0.085 | < 0.001 | 0.085 |
| cg09490124 | 7 | Body | PUS7 | 3.50E-11 | 0.11 |  | rs2517985 | 31151165 | 1.82E-08 | 9.37E-09 | 0.085 | < 0.001 | 0.085 |
| cg09490124 | 7 | Body | PUS7 | 3.50E-11 | 0.11 |  | rs746647 | 31146405 | 1.82E-08 | 9.37E-09 | 0.085 | < 0.001 | 0.085 |
| cg09468264 | 1 | NA | NA | 6.94E-12 | 0.14 |  | rs1265076 | 31145306 | 1.82E-08 | 2.67E-08 | 0.090 | < 0.001 | 0.090 |
| cg09468264 | 1 | NA | NA | 6.94E-12 | 0.14 |  | rs1265079 | 31144331 | 1.82E-08 | 2.67E-08 | 0.090 | < 0.001 | 0.090 |
| cg09468264 | 1 | NA | NA | 6.94E-12 | 0.14 |  | rs1265087 | 31142033 | 1.82E-08 | 2.67E-08 | 0.090 | < 0.001 | 0.090 |
| cg09468264 | 1 | NA | NA | 6.94E-12 | 0.14 |  | rs1265112 | 31150242 | 1.82E-08 | 2.67E-08 | 0.090 | < 0.001 | 0.090 |
| cg09468264 | 1 | NA | NA | 6.94E-12 | 0.14 |  | rs1265114 | 31149411 | 1.82E-08 | 2.67E-08 | 0.090 | < 0.001 | 0.090 |
| cg09468264 | 1 | NA | NA | 6.94E-12 | 0.14 |  | rs2517985 | 31151165 | 1.82E-08 | 2.67E-08 | 0.090 | < 0.001 | 0.090 |
| cg09468264 | 1 | NA | NA | 6.94E-12 | 0.14 |  | rs746647 | 31146405 | 1.82E-08 | 2.67E-08 | 0.090 | < 0.001 | 0.090 |
| cg09490124 | 7 | Body | PUS7 | 3.50E-11 | 0.11 |  | kgp1637562 | 31229552 | 4.22E-08 | 1.86E-08 | 0.091 | < 0.001 | 0.091 |
| cg09490124 | 7 | Body | PUS7 | 3.50E-11 | 0.11 |  | rs2844626 | 31261775 | 4.22E-08 | 1.86E-08 | 0.091 | < 0.001 | 0.091 |
| cg09043214 | 12 | Body | TNFRSF1A | 5.08E-11 | -0.11 |  | rs10484554 | 31306778 | 6.15E-09 | 2.87E-08 | 0.097 | 0.001 | 0.097 |
| cg09043214 | 12 | Body | TNFRSF1A | 5.08E-11 | -0.11 |  | rs2853952 | 31268092 | 6.15E-09 | 2.87E-08 | 0.097 | 0.002 | 0.097 |
| cg09468264 | 1 | NA | NA | 6.94E-12 | 0.14 |  | rs4406273 | 31298313 | 2.81E-09 | 2.61E-08 | 0.130 | < 0.001 | 0.130 |
| cg09061733 | 11 | TSS1500;TSS200 | SERPING1;SERPING1 | 4.08E-11 | 0.11 |  | rs10484554 | 31306778 | 6.15E-09 | 2.57E-08 | 0.141 | < 0.001 | 0.141 |
| cg09061733 | 11 | TSS1500;TSS200 | SERPING1;SERPING1 | 4.08E-11 | 0.11 |  | rs2853953 | 31267728 | 7.41E-09 | 3.24E-08 | 0.142 | < 0.001 | 0.142 |
| cg09468264 | 1 | NA | NA | 6.94E-12 | 0.14 |  | rs10484554 | 31306778 | 6.15E-09 | 1.66E-08 | 0.153 | < 0.001 | 0.153 |
| cg09468264 | 1 | NA | NA | 6.94E-12 | 0.14 |  | rs2853952 | 31268092 | 6.15E-09 | 1.66E-08 | 0.153 | < 0.001 | 0.153 |
| cg09468264 | 1 | NA | NA | 6.94E-12 | 0.14 |  | rs2844627 | 31261685 | 5.11E-08 | 5.59E-08 | 0.155 | < 0.001 | 0.155 |
| cg16085649 | 15 | Body;Body | AKAP13;AKAP13 | 8.57E-11 | 0.11 |  | rs1265076 | 31145306 | 1.82E-08 | 3.09E-08 | 0.160 | 0.001 | 0.160 |
| cg16085649 | 15 | Body;Body | AKAP13;AKAP13 | 8.57E-11 | 0.11 |  | rs1265079 | 31144331 | 1.82E-08 | 3.09E-08 | 0.160 | < 0.001 | 0.160 |
| cg16085649 | 15 | Body;Body | AKAP13;AKAP13 | 8.57E-11 | 0.11 |  | rs1265087 | 31142033 | 1.82E-08 | 3.09E-08 | 0.160 | < 0.001 | 0.160 |
| cg16085649 | 15 | Body;Body | AKAP13;AKAP13 | 8.57E-11 | 0.11 |  | rs1265112 | 31150242 | 1.82E-08 | 3.09E-08 | 0.160 | < 0.001 | 0.160 |
| cg16085649 | 15 | Body;Body | AKAP13;AKAP13 | 8.57E-11 | 0.11 |  | rs1265114 | 31149411 | 1.82E-08 | 3.09E-08 | 0.160 | < 0.001 | 0.160 |
| cg16085649 | 15 | Body;Body | AKAP13;AKAP13 | 8.57E-11 | 0.11 |  | rs2517985 | 31151165 | 1.82E-08 | 3.09E-08 | 0.160 | 0.001 | 0.160 |
| cg16085649 | 15 | Body;Body | AKAP13;AKAP13 | 8.57E-11 | 0.11 |  | rs746647 | 31146405 | 1.82E-08 | 3.09E-08 | 0.160 | < 0.001 | 0.160 |
| cg09468264 | 1 | NA | NA | 6.94E-12 | 0.14 |  | rs2853958 | 31265297 | 3.16E-09 | 7.39E-09 | 0.178 | < 0.001 | 0.178 |
| cg09490124 | 7 | Body | PUS7 | 3.50E-11 | 0.11 |  | rs1265079 | 31144331 | 1.82E-08 | 9.37E-09 | 0.182 | 0.001 | 0.182 |
| cg09468264 | 1 | NA | NA | 6.94E-12 | 0.14 |  | kgp12175980 | 31112737 | 1.02E-09 | 3.81E-08 | 0.202 | < 0.001 | 0.202 |
| cg02550308 | 16 | Body | SNTB2 | 3.32E-11 | 0.11 |  | rs1265076 | 31145306 | 1.82E-08 | 5.94E-08 | 0.206 | < 0.001 | 0.206 |
| cg02550308 | 16 | Body | SNTB2 | 3.32E-11 | 0.11 |  | rs1265079 | 31144331 | 1.82E-08 | 5.94E-08 | 0.206 | 0.001 | 0.206 |
| cg02550308 | 16 | Body | SNTB2 | 3.32E-11 | 0.11 |  | rs1265087 | 31142033 | 1.82E-08 | 5.94E-08 | 0.206 | < 0.001 | 0.206 |
| cg02550308 | 16 | Body | SNTB2 | 3.32E-11 | 0.11 |  | rs1265112 | 31150242 | 1.82E-08 | 5.94E-08 | 0.206 | < 0.001 | 0.206 |
| cg02550308 | 16 | Body | SNTB2 | 3.32E-11 | 0.11 |  | rs1265114 | 31149411 | 1.82E-08 | 5.94E-08 | 0.206 | < 0.001 | 0.206 |
| cg02550308 | 16 | Body | SNTB2 | 3.32E-11 | 0.11 |  | rs2517985 | 31151165 | 1.82E-08 | 5.94E-08 | 0.206 | < 0.001 | 0.206 |
| cg02550308 | 16 | Body | SNTB2 | 3.32E-11 | 0.11 |  | rs746647 | 31146405 | 1.82E-08 | 5.94E-08 | 0.206 | 0.002 | 0.206 |
| cg09061733 | 11 | TSS1500;TSS200 | SERPING1;SERPING1 | 4.08E-11 | 0.11 |  | rs4406273 | 31298313 | 2.81E-09 | 4.16E-08 | 0.218 | < 0.001 | 0.218 |
| cg09061733 | 11 | TSS1500;TSS200 | SERPING1;SERPING1 | 4.08E-11 | 0.11 |  | rs2853958 | 31265297 | 3.16E-09 | 2.64E-08 | 0.251 | 0.001 | 0.251 |
| cg21261487 | 18 | 5'UTR;5'UTR | MBP;MBP | 1.63E-09 | -0.10 |  | rs2853952 | 31268092 | 6.15E-09 | 5.56E-08 | 0.209 | 0.512 | 0.512 |
| cg09061733 | 11 | TSS1500;TSS200 | SERPING1;SERPING1 | 4.08E-11 | 0.11 |  | rs2245822 | 31263023 | 4.17E-09 | 4.33E-08 | 0.684 | 0.002 | 0.684 |
| cg00288598 | 8 | Body;Body | EIF2C2;EIF2C2 | 6.51E-12 | -0.18 |  | rs2853958 | 31265297 | 3.16E-09 | 5.95E-08 | 0.694 | 0.001 | 0.694 |
| cg27571329 | 1 | Body;Body | FGGY;FGGY | 7.69E-11 | 0.12 |  | rs1265114 | 31149411 | 1.82E-08 | 4.83E-08 | 0.723 | 0.136 | 0.723 |
| cg27571329 | 1 | Body;Body | FGGY;FGGY | 7.69E-11 | 0.12 |  | rs1265076 | 31145306 | 1.82E-08 | 4.83E-08 | 0.003 | 0.783 | 0.783 |
| cg21116284 | 1 | Body | TGFBR3 | 1.06E-09 | 0.11 |  | kgp1637562 | 31229552 | 4.22E-08 | 2.71E-08 | 0.120 | 0.789 | 0.789 |
| cg19430423 | 19 | Body | CYP2S1 | 6.71E-13 | -0.16 |  | kgp12175980 | 31112737 | 1.02E-09 | 5.37E-08 | 0.806 | 0.53 | 0.806 |
| cg27571329 | 1 | Body;Body | FGGY;FGGY | 7.69E-11 | 0.12 |  | rs746647 | 31146405 | 1.82E-08 | 4.83E-08 | 0.822 | 0.516 | 0.822 |
| cg21653149 | 17 | Body;Body | ANKFY1;ANKFY1 | 9.88E-11 | 0.12 |  | rs1265087 | 31142033 | 1.82E-08 | 5.79E-08 | 0.822 | 0.564 | 0.822 |
| cg27571329 | 1 | Body;Body | FGGY;FGGY | 7.69E-11 | 0.12 |  | rs1265079 | 31144331 | 1.82E-08 | 4.83E-08 | 0.822 | 0.551 | 0.822 |
| cg19430423 | 19 | Body | CYP2S1 | 6.71E-13 | -0.16 |  | rs2853952 | 31268092 | 6.15E-09 | 2.59E-08 | 0.854 | 0.293 | 0.854 |
| cg21653149 | 17 | Body;Body | ANKFY1;ANKFY1 | 9.88E-11 | 0.12 |  | rs746647 | 31146405 | 1.82E-08 | 5.79E-08 | 0.859 | 0.492 | 0.859 |
| cg20335425 | 1 | 5'UTR | S100A8 | 6.94E-12 | -0.12 |  | rs2853953 | 31267728 | 7.41E-09 | 4.88E-08 | 0.146 | 0.871 | 0.871 |
| cg20335425 | 1 | 5'UTR | S100A8 | 6.94E-12 | -0.12 |  | rs10484554 | 31306778 | 6.15E-09 | 4.03E-08 | NA | < 0.001 | NA |
| cg21653149 | 17 | Body;Body | ANKFY1;ANKFY1 | 9.88E-11 | 0.12 |  | rs1265076 | 31145306 | 1.82E-08 | 5.79E-08 | NA | < 0.001 | NA |
| cg21653149 | 17 | Body;Body | ANKFY1;ANKFY1 | 9.88E-11 | 0.12 |  | rs1265079 | 31144331 | 1.82E-08 | 5.79E-08 | NA | < 0.001 | NA |
| cg27571329 | 1 | Body;Body | FGGY;FGGY | 7.69E-11 | 0.12 |  | rs1265087 | 31142033 | 1.82E-08 | 4.83E-08 | NA | 0.532 | NA |
| cg27571329 | 1 | Body;Body | FGGY;FGGY | 7.69E-11 | 0.12 |  | rs1265112 | 31150242 | 1.82E-08 | 4.83E-08 | NA | 0.892 | NA |
| cg21653149 | 17 | Body;Body | ANKFY1;ANKFY1 | 9.88E-11 | 0.12 |  | rs1265112 | 31150242 | 1.82E-08 | 5.79E-08 | NA | 0.54 | NA |
| cg21653149 | 17 | Body;Body | ANKFY1;ANKFY1 | 9.88E-11 | 0.12 |  | rs1265114 | 31149411 | 1.82E-08 | 5.79E-08 | NA | < 0.001 | NA |
| cg19430423 | 19 | Body | CYP2S1 | 6.71E-13 | -0.16 |  | rs2245822 | 31263023 | 4.17E-09 | 3.42E-08 | NA | 0.284 | NA |
| cg27571329 | 1 | Body;Body | FGGY;FGGY | 7.69E-11 | 0.12 |  | rs2517985 | 31151165 | 1.82E-08 | 4.83E-08 | NA | 0.569 | NA |
| cg21653149 | 17 | Body;Body | ANKFY1;ANKFY1 | 9.88E-11 | 0.12 |  | rs2517985 | 31151165 | 1.82E-08 | 5.79E-08 | NA | < 0.001 | NA |
| cg21116284 | 1 | Body | TGFBR3 | 1.06E-09 | 0.11 |  | rs2844626 | 31261775 | 4.22E-08 | 2.71E-08 | NA | < 0.001 | NA |
| cg20335425 | 1 | 5'UTR | S100A8 | 6.94E-12 | -0.12 |  | rs2853952 | 31268092 | 6.15E-09 | 4.03E-08 | NA | 0.949 | NA |
| cg21653149 | 17 | Body;Body | ANKFY1;ANKFY1 | 9.88E-11 | 0.12 |  | rs2853958 | 31265297 | 3.16E-09 | 2.28E-08 | NA | < 0.001 | NA |
| cg19430423 | 19 | Body | CYP2S1 | 6.71E-13 | -0.16 |  | rs4406273 | 31298313 | 2.81E-09 | 4.38E-08 | NA | < 0.001 | NA |

Gene name: according to HUGO Gene Nomenclature Committee. Gene_context: location of the gene associated CpG-site(s) with respect to the gene context. NA: not available.
Illumina ID is arranged by significance rank of CIT test. CIT *P*: the maximum of the component *P*-values for an omnibus test. *P*_CIT_ < 0.05 is highlighted in grey.

**Table S5. Cross validation between Illumina and Sequenom platform**

| **Gene name** |  | **Sequenom** | |  | **Illumina** | |  | **Spearman Correlation** | |
| --- | --- | --- | --- | --- | --- | --- | --- | --- | --- |
|  |  | **Mean.beta** | **Std err** |  | **Mean.beta** | **Std err** |  | **Pho** | ***P*-value** |
| cg00288598 |  | 0.48 | 0.10 |  | 0.48 | 0.11 |  | 0.90 | 2.19E-09 |
| cg09468264 |  | 0.32 | 0.10 |  | 0.31 | 0.11 |  | 0.78 | 7.21E-06 |
| cg13086983 |  | 0.71 | 0.07 |  | 0.76 | 0.06 |  | 0.82 | 8.20E-07 |
| cg13375463 |  | 0.64 | 0.08 |  | 0.68 | 0.07 |  | 0.80 | 2.43E-06 |
| cg19430423 |  | 0.51 | 0.10 |  | 0.50 | 0.07 |  | 0.84 | 3.34E-07 |
| cg20335425 |  | 0.58 | 0.11 |  | 0.57 | 0.10 |  | 0.89 | 5.03E-09 |
| cg22724943 |  | 0.44 | 0.06 |  | 0.45 | 0.06 |  | 0.89 | 7.00E-09 |
| cg24216770 |  | 0.52 | 0.12 |  | 0.54 | 0.10 |  | 0.90 | 3.30E-09 |
| cg26756782 |  | 0.67 | 0.10 |  | 0.69 | 0.08 |  | 0.70 | 2.12E-04 |

9 CpGs methylation level of 24 PP samples from Illumuna were evaluated by Epitype technology. All the speram rank correlation pho reahced the significance level (P < 0.01). Mean.beta: The average beta for each probe. Std err: stardard error. Pho, P-value: Speraman correlation coefficient and *P* between Illumina and Sequenom platform for each probe.
