# Supplementary figures and images for: Epigenome-wide association data implicates DNA methylation-mediated genetic risk in psoriasis
Source: Clin Epigenetics. 2016 Dec 5;8:131. doi: 10.1186/s13148-016-0297-z (PMC5139011; doi:10.1186/s13148-016-0297-z)

**Figure S1. Genomic annotation for methylation probes.**


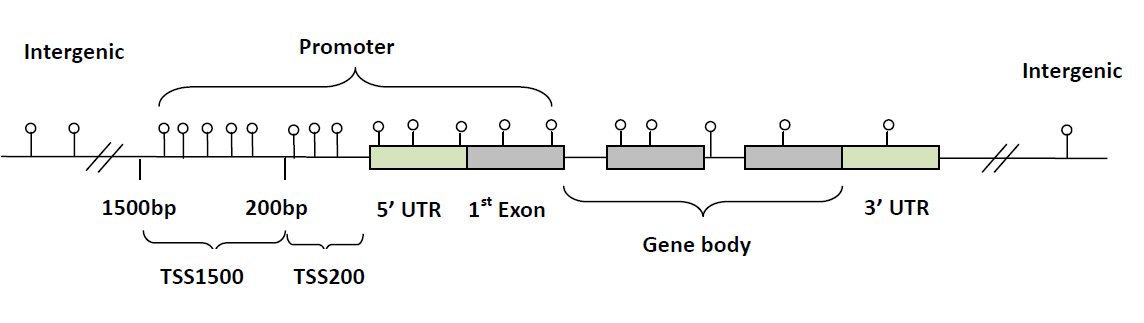

Supplement: Additional file 2: — Figure S1. Genomic annotation for methylation probes. Refseq genes annotations were from the University of California, Santa Cruz (UCSC) hg18 reference genome [National Center for Biotechnology Information (NCBI) Reference Sequence Database Release 37]. CpG sites located in or near Refseq genes are separated into TSS1500 (1500 bp upstream of the transcription start site), TSS200, 5′ UTR, first exon, gene body, and 3′ UTR regions. Intergenic probes refer to methylation sites not mapping to any of the other categories. The ambiguous regions include sites that fell into two or more different categories. The promoter region includes methylation sites located in TSS1500, TSS200, 5′ UTR, and first exon. (DOC 92 kb) [file 13148_2016_297_MOESM2_ESM.doc]
